# Supplementary material for: Management and Prognosis of Cardiac Metastatic Merkel Cell Carcinoma: A Case–Control Study and Literature Review
Source: Cancers (Basel). 2022 Nov 30;14(23):5914. doi: 10.3390/cancers14235914 (PMC9741306; doi:10.3390/cancers14235914)
Supplement: Supplementary file 1 [file cancers-14-05914-s001.zip › cancers-1991981-supplementary.pdf]

Supplementary Material:
Selection of the matched reference cohort of non-cardiac mMCC patients

To explore whether patients with cardiac mMCC have worse OS compared to patients with non-cardiac mMCC, we selected a reference cohort with non-cardiac mMCC from the prospective MCC observational registry. The reference cohort was matched to the cardiac mMCC patients using the following covariates that may influence OS, including immune suppression status, age (± 10 years), sex, disease status of stage IV at initial diagnosis, and a number of prior metastatic episodes. The number of prior metastatic episodes counted the initial diagnosis if stage IV, and each time a distant recurrence or progression was documented, regardless of the number of metastatic sites. For example, if metastases were identified at two different sites on the same scan, that was considered a single episode for the purposes of matching patients.

As another example, consider the following hypothetical matching: there is a cardiac mMCC patient whose first cardiac metastasis was the second metastatic episode, meaning that the patient had local or regional disease at diagnosis, a subsequent non-cardiac distant recurrence that was treated, and finally a subsequent distant recurrence to the heart. A matching patient would have the same covariate values as listed above and would also have had initially local or regional disease at diagnosis followed by two non-cardiac distant recurrences documented at different times. The time of the first cardiac metastasis would be used as the time origin for the cardiac mMCC patient, and the time of the second distant recurrence would be used as the time origin for the matching non-cardiac mMCC to make their outcomes comparable.

Prior to matching, patients were excluded if any of the required data were missing: consent date, survival follow-up (date of death if died or last follow-up otherwise), initial stage, recurrence follow-up if stage I-III, date of birth, sex, immunosuppression status, or enrollment >180 days after the matching metastatic episode. For each cardiac mMCC patient, all non-cardiac mMCC patients who matched that cardiac mMCC patient were selected for the reference cohort. The reference cohort patients were then re-weighted so that each group of matches for each cardiac mMCC patient had the same weight when estimating the reference OS curve (Cole SR and Hernan MA. Adjusted survival curves with inverse probability weights. Computer Methods and Programs in Biomedicine. 2004. 75(1):45-9. PMID: 15158046). For example, if one cardiac mMCC patient matched 10 non-cardiac mMCC patients, each of those non-cardiac mMCC patients would be given a weight of one-tenth, so their total weight was equivalent to a single patient. Patients were weighted so the number of matches would not skew the OS estimates, and multiple matches were included rather than a single random match to order to increase the overall sample size and achieve a narrower confidence interval for the OS of the reference cohort.
